# Gene expression of prostaglandin EP4 receptor in three canine carcinomas

**DOI:** 10.1186/s12917-020-02431-2

**Published:** 2020-06-22

**Authors:** Margaret L. Musser, Austin K. Viall, Rachel L. Phillips, Jesse M. Hostetter, Chad M. Johannes

**Affiliations:** 1 Department of Veterinary Clinical Sciences, Iowa State University College of Veterinary Medicine, Ames, IA USA; 2 Department of Veterinary Pathology, Iowa State University College of Veterinary Medicine, Ames, IA USA; 3 Present address: University of Georgia College of Veterinary Medicine, 501 D.W. Brooks Drive, Athens, GA 30602 USA

**Keywords:** Cancer, Canine, Carcinoma, Cyclooxygenase enzyme 2, EP4 receptor, Inflammation, RNAscope®

## Abstract

**Background:**

Chronic inflammation mediated by the cyclooxygenase enzymes, specifically their product prostaglandin E2 (PGE2), can result in the development of cancer. PGE2 promotes cell proliferation, apoptosis, and angiogenesis through interaction with its specific receptors (EP1 receptor - EP4 receptor [EP1R-EP4R]). In multiple human cancers, the expression of EP4R is associated with the development of malignancy and a poor prognosis. The expression of EP4R has not yet been evaluated in canine tumors. The aim of this study was to characterize the mRNA gene expression of EP4R (*ptger4*) in canine squamous cell carcinoma (SCC), apocrine gland anal sac adenocarcinoma (AGASACA), and transitional cell carcinoma (TCC). Archived tumor samples of canine cutaneous SCC (*n* = 9), AGASACA (*n* = 9), and TCC (*n* = 9), and matched archived normal tissue controls were evaluated for mRNA expression of canine EP4R using RNA in situ hybridization (RNAscope®). Quantification of RNAscope® signals in tissue sections was completed with an advanced digital pathology image analysis system (HALO). Data was expressed as copy number, H-index, and percent tumor cell expression of EP4R.

**Results:**

In all canine SCC, AGASACA, and TCC samples evaluated, strong universal positive expression of EP4R was identified. For SCC and AGASACA, mRNA EP4R expression was statistically higher than that of their respective normal tissues. The TCC tissues displayed significantly less mRNA EP4R expression when compared to normal bladder mucosa.

**Conclusions:**

These results confirm the mRNA expression of canine EP4R in all tumor types evaluated, with SCC and AGASACA displaying the highest expression, and TCC displaying the lowest expression. This study also represents the first reported veterinary evaluation of EP4R expression using the novel in situ hybridization technique, RNAscope®.

## Background

Chronic immune activation and subsequent inflammation triggered by an infectious cause, foreign antigen, or carcinogenic stimulant, can promote the development of cancer [1]. Exposure to an inciting cause results in the upregulation of non-specific pro-inflammatory cytokines and enzymes, the most important of which is the cyclooxygenase enzyme 2 (COX-2). This enzyme stimulates angiogenesis, inhibits apoptosis, and promotes cell proliferation and motility, supporting the promotion and progression of cancer [1]. There is substantial evidence that COX-2 expression promotes tumor development [2] and progression in multiple human cancers including cutaneous squamous cell carcinoma [3–6], urothelial carcinoma [7], and colorectal carcinoma [8].

The primary function of COX-2 is to convert arachidonic acid to prostaglandins (PGs). The most active and predominant product in this cascade is prostaglandin E2 (PGE2), which drives many normal physiological functions including inflammation, modulation of gastrointestinal mucosa, bone healing, and vasodilation [9]. In tumor tissues, high levels of PGE2 stimulate cell proliferation, apoptosis, and angiogenesis [10]. High levels of PGE2 also suppress antitumor immunity in the tumor microenvironment, allowing progression of disease [2, 11]. This is accomplished through multiple mechanisms including inhibition of dendritic cell recruitment, inhibition of natural killer cells, decreased infiltration of cytotoxic T-lymphocytes, activation of myeloid-derived suppressor cells, and increased tumor-infiltrating T-regulatory cells [2, 11, 12].

The physiological activities of PGE2 are mediated through its four currently recognized receptors (EP1 receptor to EP4 receptor [EP1R-EP4R]) on the surface of the target cells [13]. Concurrent with the upregulation of COX-2 and thus PGE2 in multiple cancer types, it has also been found that the EP receptors are differentially expressed and associated with the development of malignancy and poor prognosis in several human cancers [2, 14]. In humans, EP1R has been shown to activate signaling cascades mediating cell migration and invasion, but is also associated with improved outcome through anti-metastatic functions. Reasons for these contrasting findings are unclear, but may be due to the tissue-specific functional activities of the EP receptors [15]. In specific malignancies, activation of EP2R most commonly induces angiogenesis and suppression of the antitumor immune response [15]. The role of EP3R is unclear, with multiple conflicting studies [15]. The EP4 receptor has the most robust data regarding its role in tumorigenesis. Its activation promotes the development of a pro-tumorigenic immune response, and has been shown to stimulate tumor cell migration, proliferation and metastasis [15]. EP4R has been shown to have increased expression in human cutaneous squamous cell carcinoma [16], and to be the most abundant EP receptor subtype in human urinary tract transitional cell carcinoma [17], and colorectal cancer [18], among others [15].

In addition to EP receptor overexpression and thus upregulation of various signaling cascades associated with tumorigenesis, PGE2 modification of the tumor microenvironment and evasion of the immune system is regulated through the EP receptors. Specifically, signaling through EP4R promotes immune evasion of cancer cells through suppression of natural killer cells and cytotoxic T-lymphocytes, and activation of myeloid derived suppressor cells and T-regulatory cells [15]. These changes in the microenvironment disrupt the concept of the cancer-immunity cycle (C-IC) proposed by Chen and Mellman [19]. The C-IC model describes the series of stepwise events required to initiate an anticancer immune response, reliant upon the interactions of tumor-derived antigens, effector cytotoxic T-lymphocytes, dendritic cells, and tumor cells themselves [19]. EP4R antagonists can reactivate antitumor immunity, stimulating the C-IC response by restoring the PGE2-mediated dysfunctions in the antitumor immune response [11].

Several human studies have investigated the therapeutic impact of COX-2 inhibitors for the prevention of tumorigenesis [20]. However, there is mounting evidence that carcinogenesis and tumor progression is regulated specifically by EP receptors, and thus their targeted blockade may offer therapeutic advantage [15, 17]. In fact, antagonism of the EP1 and EP4 receptors has resulted in suppression of human tumor development and progression across tumor type including tongue squamous cell carcinoma, skin tumors, and colonic carcinoma. Due to the lack of selective EP2R antagonists, targeting this receptor is less desirable and successful. Similarly, EP3R antagonists have not been successful clinically [9, 15]. Detailed data regarding expression of each EP receptor in various malignancies is necessary to fully understand the impact of COX-2 and EP receptor inhibition, and to design effective treatment and prevention strategies. These investigations are in the early stages in human medicine, and are limited on the veterinary side.

Studies in dogs have revealed expression of COX-2 in various tumor types including cutaneous squamous cell carcinoma [21, 22], urinary transitional cell carcinoma [23], apocrine gland anal sac adenocarcinoma (AGASACA) [24], and mammary carcinoma [25], among others [26]. Expression of EP2R has been confirmed in canine mammary carcinoma and osteosarcoma [25, 27]. Characterization of the canine EP4R has been completed [28], and only recently has the positive gene expression of EP4R in canine osteosarcoma been reported [29]. Expression of EP4R in other canine cancers has not been evaluated [25].

Multiple clinical, experimental, and epidemiological studies indicate that COX-2 inhibitors show therapeutic potential in canine malignancies [30, 31]. Blockade of COX-2 by non-steroidal anti-inflammatory drugs (NSAIDs), alone or in conjunction with chemotherapy, leads to tumor control and prolonged survival in several canine cancers expressing COX-2 including squamous cell carcinoma and urinary transitional cell carcinoma [26, 32]. Clinically, NSAIDs are often used for the treatment of AGASACA due to increased expression of COX-2 [24]. However, NSAIDs are not specific and attenuate the production of prostanoids other than PGE2 that are important in homeostasis [33]. Thus, alternative treatment options that are more specific, such as an EP receptor antagonist, may provide an anti-cancer benefit with an improved safety profile.

Based on the human literature, and the sparse canine literature, it appears reasonable to hypothesize that expression of EP4R may be present and play a role in the development and progression of common canine cancers. In addition, should EP4R be expressed, therapeutic blockade with the FDA-approved canine EP4R antagonist (grapiprant) may prove to be clinically beneficial. This study aims to be a pilot analysis of the gene expression of EP4R in three common, aggressive canine carcinomas that are typically treated with a combination of surgery (if possible), chemotherapy, and NSAIDs (cutaneous squamous cell carcinoma [SCC], AGASACA, and transitional cell carcinoma of the urinary bladder [TCC]). These cancers were chosen specifically due to their frequency within the canine population [34], expression of COX-2, and common clinical use and response to NSAID treatment. The EP4 receptor was targeted for investigation as it appears to play a major role in the development of human malignancy [15], and a commercially available, highly specific EP4R antagonist is available in veterinary medicine (grapiprant). This study represents a proof-of-concept and first step in analysis of EP4R expression in several canine malignancies.

## Results

**Fig. 1:**
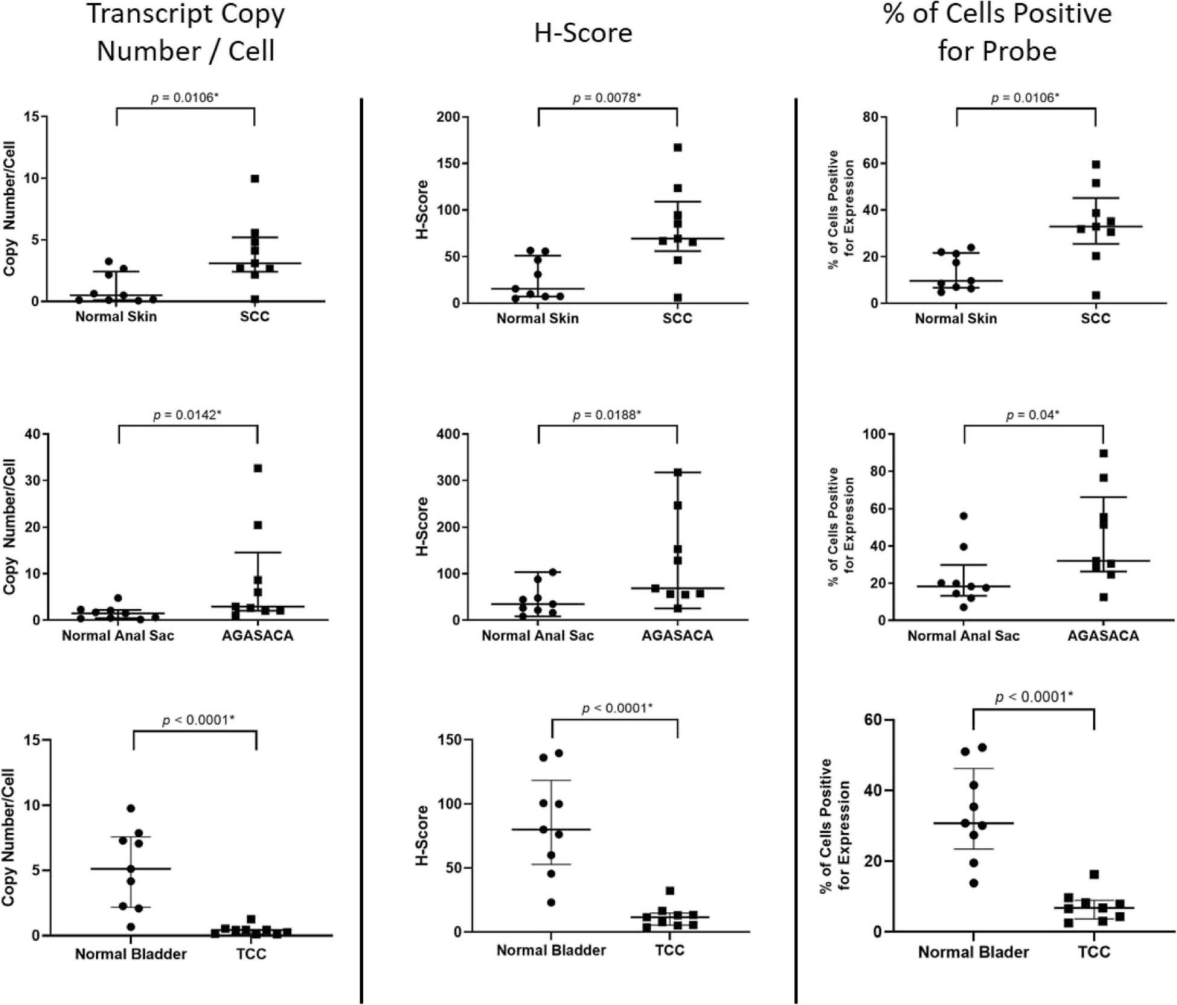
EP4R mRNA Expression Metrics in Squamous Cell Carcinoma vs. Normal Skin, Apocrine Gland Anal Sac Adenocarcinoma vs. Normal Anal Sac, and Transitional Cell Carcinoma vs. Normal Bladder. Data presented as median and interquartile range, with all data points visualized; * denotes differences was statistically significant. SCC: Squamous Cell Carcinoma; AGASACA: Apocrine Gland Anal Sac Adenocarcinoma; TCC: Transitional Cell Carcinoma

**Fig. 2:**
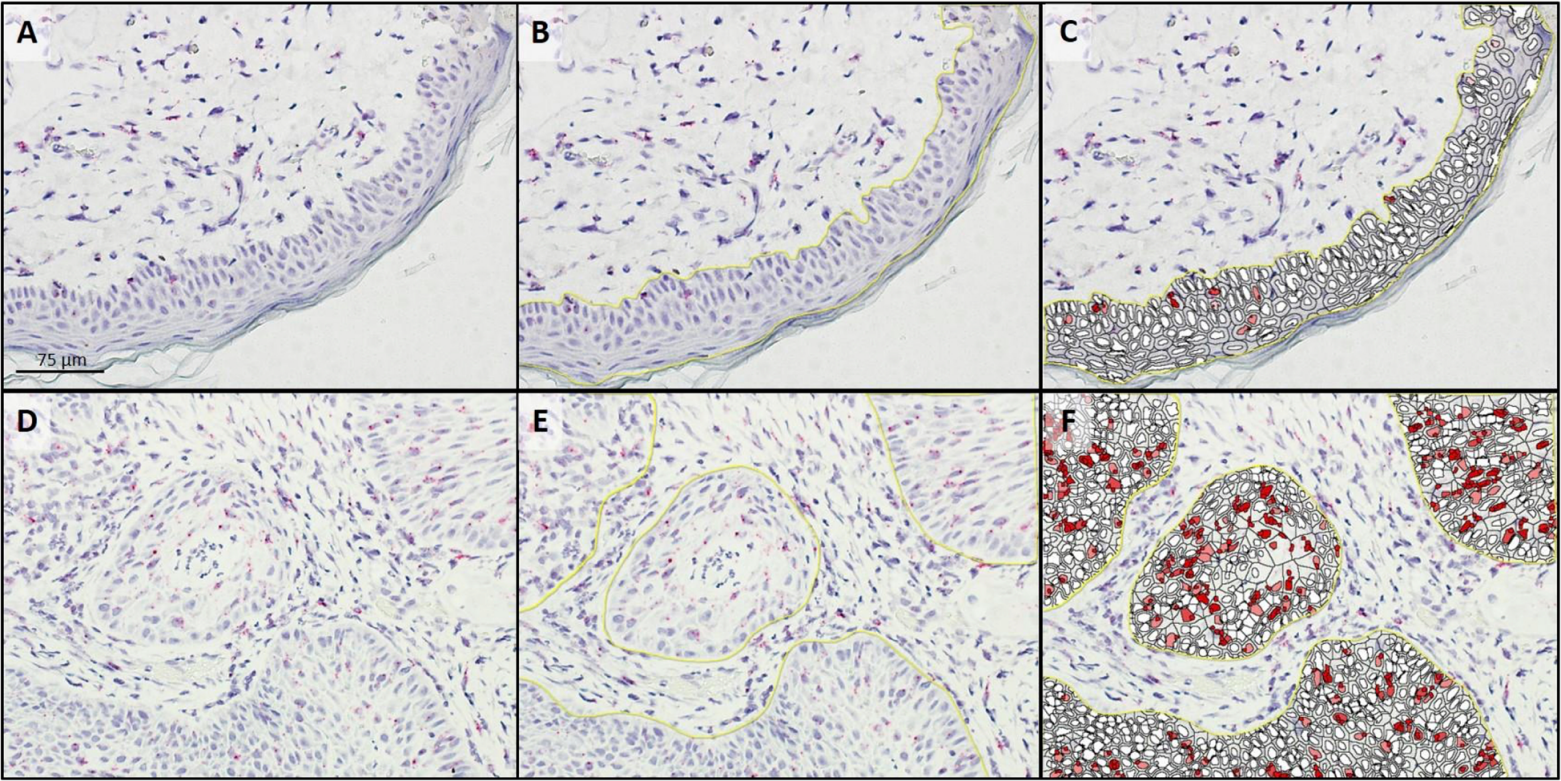
Analysis of *ptger4* transcription in a section of normal skin (**a**-**c**) and squamous cell carcinoma (**d**-**f**) following RNAscope® mRNA in situ hybridization with hematoxylin counter stain. **a** & **d**: Native photomicrograph to be analyzed for copy number/cell, H-Score, and percentage transcript expression using HALO software with RNAscope® Modules. **b** & **e**: Target cells were manually gated (yellow line) for analysis. **c** & **f**: HALO generated probe markup from which copy number/cell, H-Score, and percentage transcript expression are calculated

Fourteen SCC samples and 17 normal skin samples were examined to identify 9 samples of each (tumor and normal tissues; Table 1) that had sufficient residual mRNA for *ptger4* expression analysis. All 9 SCC and 9 normal skin samples with sufficient mRNA expressed EP4R mRNA. The median copy number per cell, H-score, and percent probe positive scores were statistically higher in the SCC samples compared to the normal skin samples (Table 2; Figs. 1 and 2).

**Table 1:** Clinical characteristics of biopsy samples evaluated for EP4R

Case	Age	Gender and neuter status	Breed	Case	Age	Gender and neuter status	Breed
**SCC tumor tissues**				**Normal skin tissues**			
1	NR	FS	Labrador Retriever	1	NR	FS	Collie
2	1	MN	German Shepherd Dog	2	NR	MN	Yorkshire Terrier
3	6	FS	Mixed Breed	3	3	FS	Chihuahua
4	10	FS	Beagle	4	3	MN	Mixed Breed
5	10	FS	English Springer Spaniel	5	5	MI	Red Bone Hound
6	10	MN	Mixed Breed	6	6	MN	Boxer
7	11	FS	Mastiff	7	9	MN	Bichon Frise
8	13	MN	Mixed Breed	8	9	FS	Labrador Retriever
9	13	MN	Norwich Terrier	9	12	FS	German Shorthaired Pointer
**AGASACA tumor tissues**				**Normal anal sac tissues**			
1	6	MI	Akita	1	2	FS	Mixed Breed
2	8	FS	Labrador Retriever	2	3	MN	Chow-Chow
3	8	MN	Mixed Breed	3	4	FS	Pitbull
4	9	MN	Brussels Griffon	4	6	MN	Mixed Breed
5	9	MN	Mixed Breed	5	8	FS	Mixed Breed
6	10	FS	Cavalier King Charles Spaniel	6	9	FS	Cocker Spaniel
7	10	FS	Labrador Retriever	7	10	FS	Mixed Breed
8	11	FS	Mixed Breed	8	11	FS	Mixed Breed
9	14	FS	Labrador Retriever	9	12	FS	Beagle
**TCC tumor tissues**				**Normal bladder tissues**			
1	NR	MI	Beagle	1	4	MN	Goldendoodle
2	4	FS	Pitbull	2	5	FI	German Shorthair Pointer
3	6	FS	Golden Retriever	3	5	MN	Weimaraner
4	9	MN	Great Dane	4	8	MN	Yorkshire Terrier
5	10	MN	Sheltie	5	9	MN	Weimaraner
6	11	MN	Golden Retriever	6	11	MN	Australian Shepherd
7	13	MN	Mixed Breed	7	12	MN	Mixed Breed
8	14	FS	Chihuahua	8	13	FS	Cocker Spaniel
9	14	MN	West Highland White Terrier	9	15	FS	Toy Poodle

*AGASACA* Apocrine Gland Anal Sac Adenocarcinoma, *EP4R* EP4 receptor, *FI* Female intact, *FS* Female spayed, *MI* Male intact, *MN* Male neutered, *NR* Not reported, *SCC* Squamous Cell Carcinoma, *TCC* Transitional Cell Carcinoma

**Table 2:** EP4R mRNA expression levels in three canine malignancies

Tissue^**a**^	Median copy number/Cell	Median H-score	Median % probe positive
SCC	3.1 (range: 0.2–10.0)	69.51 (range: 6.001–167.2)	32.45 (range: 3.485–59.54)
Skin	0.5 (range: 0.1–3.3)	15.56 (range: 5.071–56.80)	9.67 (range: 4.812–24.01)
*p-value*	0.0106*	0.0078*	0.0106*
AGASACA	2.9 (range: 1.1–32.64)	68.79 (range: 25.62–317.4)	32.06 (range: 12.55–89.59)
Anal Sac	1.5 (range: 0.2–4.8)	34.76 (range: 8.439–103.4)	18.28 (range: 7.118–56.19)
*p-value*	0.0142*	0.0188*	0.0400*
TCC	0.4 (range: 0.1–1.3)	11.62 (range: 3.671–32.23)	6.80 (range: 2.505–16.25)
Bladder	5.1 (range: 0.7–9.8)	80.00 (range: 23.06–139.6)	30.78 (range: 13.84–52.19)
*p-value*	< 0.0001*	< 0.0001*	< 0.0001*

*SCC* Squamous Cell Carcinoma, *AGASACA* Apocrine Gland Anal Sac Adenocarcinoma, *TCC* Transitional Cell Carcinoma

^**a**^Non-normally distributed data was evaluated with the Mann Whitney test between neoplastic cells and corresponding normal tissue type; the median and range are reported

*Statistically significant

**Fig. 3:**
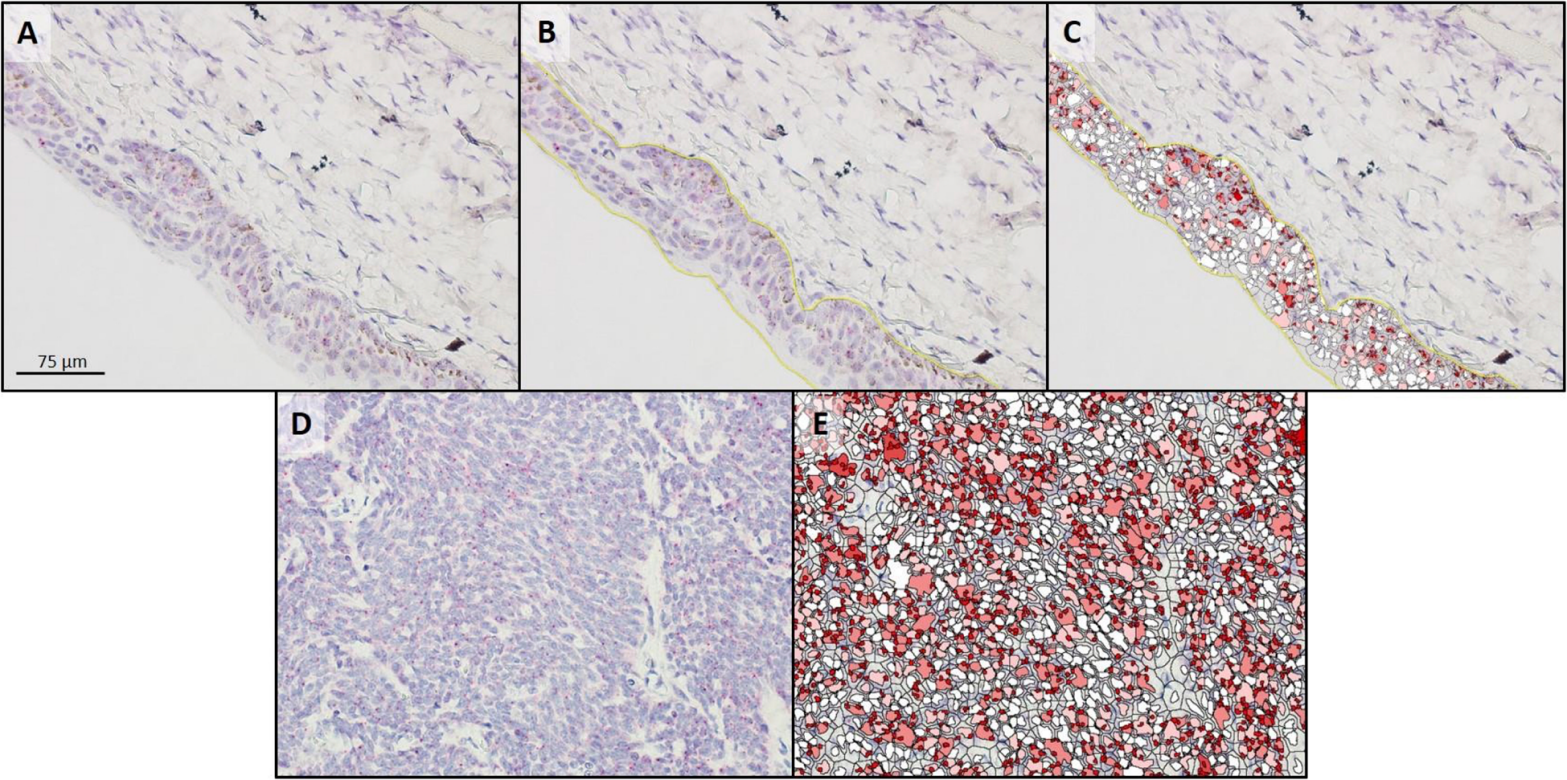
Analysis of *ptger4* transcription in a section of normal anal sac (**a**-**c**) and apocrine gland anal sac adenocarcinoma (**d**, **e**) following RNAscope® mRNA in situ hybridization with hematoxylin counter stain. **a** & **d**: Native photomicrograph to be analyzed for copy number/cell, H-Score, and percentage transcript expression using HALO software with RNAscope® Modules. **b**: Target cells were manually gated (yellow line) for analysis; no gated cells for tumor image since all displayed cells were neoplastic. **c** & **e**: HALO generated probe markup from which copy number/cell, H-Score, and percentage transcript expression are calculated

Fourteen AGASACA and 13 normal anal sac samples were examined to identify 9 samples of each (tumor and normal tissues; Table 1) that had sufficient residual mRNA for *ptger4* expression analysis. All 9 AGASACA and 9 normal anal sac samples with sufficient mRNA expressed EP4R mRNA. The median copy number per cell, H-score, and percent probe positive scores were statistically higher in the AGASACA samples compared to the normal anal gland (Table 2; Figs. 1 and 3).

**Fig. 4:**
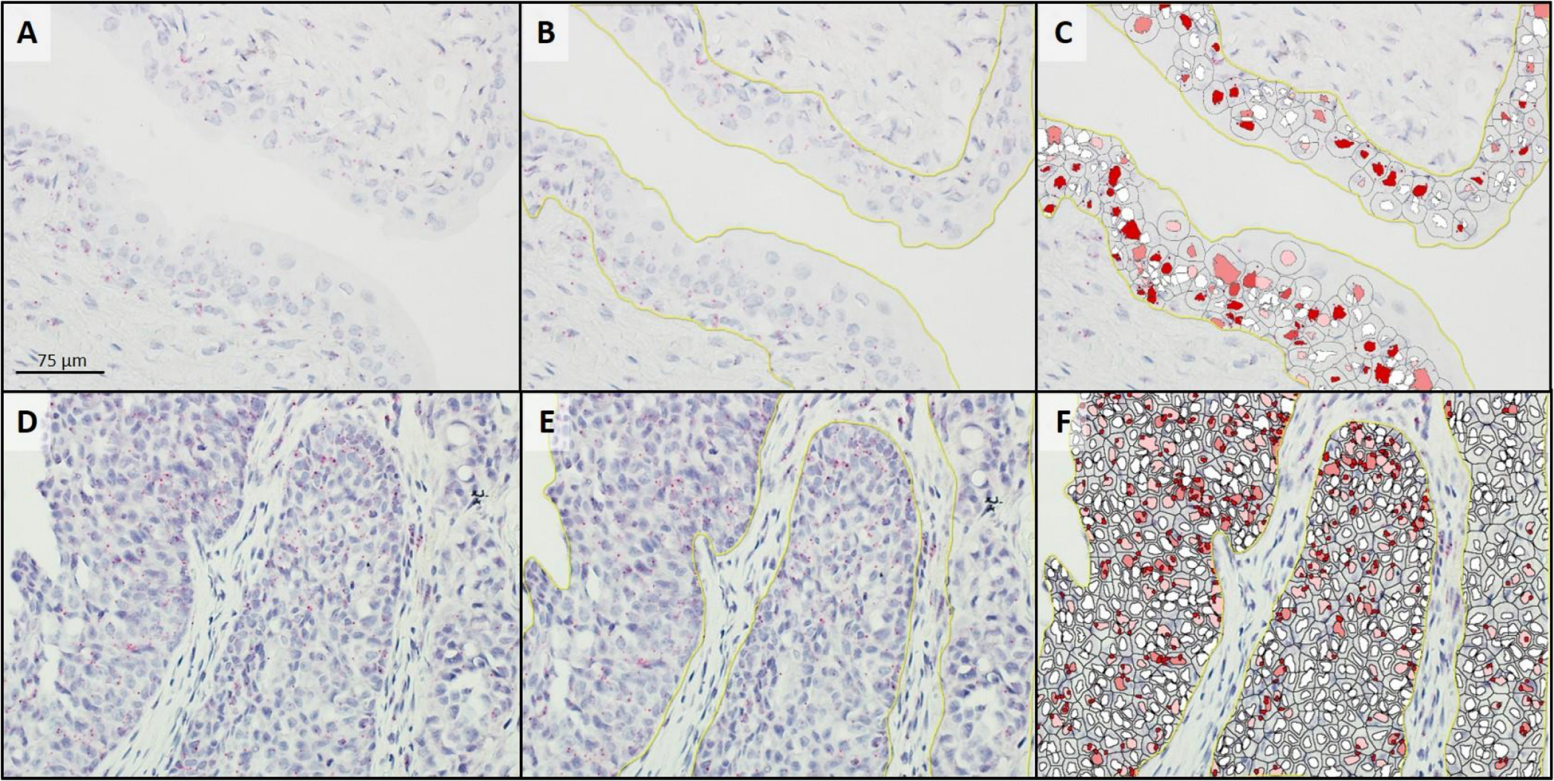
Analysis of *ptger4* transcription in a section of normal bladder (**a**-**c**) and transitional cell carcinoma (**d**-**f**) following RNAscope® mRNA in situ hybridization with hematoxylin counter stain. **a** & **d**: Native photomicrograph to be analyzed for copy number/cell, H-Score, and percentage transcript expression using HALO software with RNAscope® Modules. **b** & **e**: Target cells were manually gated (yellow line) for analysis. **c** & **f**: HALO generated probe markup from which copy number/cell, H-Score, and percentage transcript expression are calculated

Twelve TCC and 13 normal bladder samples were examined to identify 9 samples of each (tumor and normal tissues; Table 1) that had sufficient residual mRNA for *ptger4* expression analysis. All 9 samples of both the tumor and normal tissues with sufficient mRNA expressed EP4R mRNA. The median copy number per cell, H-score, and percent probe positive scores were statistically different between the TCC samples and the normal bladder samples (Table 2; Fig. 1). However, in contrast to the SCC and AGASACA tissues, it was found that the TCC samples had statistically less EP4R mRNA when compared to the normal bladder (Fig. 4).

The copy number per cell, H-score, and percent probe positive scores for each of the three tumor types were variable, with SCC and AGASACA displaying the highest expression, and TCC displaying the lowest expression (Table 2; Fig. 1).

## Discussion

Positive expression of EP4R mRNA was found in three different canine carcinomas and matching control tissues. With the exception of TCC, each tumor had statistically higher expression of EP4R mRNA when compared to the matched normal tissue. These preliminary results suggest that EP4R expression may play a role in the pathogenesis and development of these tumors.

The expression of EP receptors are associated with the development of malignancy and poor prognosis in several human cancers [14]. In particular, EP4R has increased expression in human cutaneous squamous cell carcinoma [16], and is the most abundant EP receptor subtype in multiple human malignancies [15] including urinary tract transitional cell carcinoma [17] and colorectal carcinoma [18]. Additionally, carcinogenesis and tumor progression are regulated in part by EP receptors [17]. The current study suggests that this may be also true in canine cancer.

In canine cutaneous SCC, EP4R mRNA expression was detected and was significantly higher in malignant tissue compared to normal skin. Canine cutaneous SCC frequently develops in areas of light pigmentation and sparse fur coat, most commonly on the ventral abdomen, due to exposure to ultraviolet light [35]. Increased expression of COX-2 has also been identified as a possible driver of carcinogenesis of canine cutaneous SCC [21, 22]. In humans, upregulated COX-2 expression is found following acute ultraviolet B (UVB) exposure and in UVB-induced cutaneous SCC [36]. In addition, in murine models of UVB-induced SCC and naturally occurring human UVB-induced cutaneous SCC, enhanced expression of EP4R is present in cancerous SCC compared to adjacent non-tumor-bearing skin [16]. The anatomical location of all canine skin samples examined for EP4R expression was not known. At least 5 of the SCC samples and at least 1 of the normal tissue samples were from areas of increased UV exposure. These data suggest that canine and human UVB-induced SCC have similar pathogeneses and may be influenced by upregulation of COX-2, PGE2, and increased expression of EP4R.

Similarly, canine AGASACA tissue had significantly higher expression of EP4R. Anal gland adenocarcinoma is rare in humans [37] and the impact of COX-2 or EP4R expression in the development of this tumor has not been evaluated. Increased expression of COX-2 has been previously shown in canine AGASACA [24]. The results of this study suggest that COX-2 effects may be mediated by EP4R, indicating therapeutic blockade of EP4R may be a reasonable treatment strategy in this cancer.

In canine TCC, although EP4R expression was present, it was found to be statistically lower than the expression of EP4R in the normal bladder. This unexpected finding is similar to the pattern seen in some evaluations of human colorectal carcinoma, where the high cellular density of advanced tumors induces the expression of hypoxia inducible factor 1-alpha (HIF-1⍺), which appears to have a negative feedback effect on the expression of EP4R while increasing the expression of EP3R [38]. Conversely, other colorectal studies have shown increased EP4R mRNA expression with progressive invasiveness and tumor grade [39, 40]. Divergent EP4R expression has also been observed in a murine model of human high-grade invasive bladder cancer: early in the course of disease, expression of mRNA for EP4R is increased, but later in the course of disease, EP4R expression decreased [41]. If HIF-1⍺ controls an EP4R negative feedback loop, one would expect all tumors with increased HIF-1⍺ to display this pattern. This was not seen in the current study, despite the fact that AGASACA has been shown to express HIF-1⍺ [42]; the expression of HIF-1⍺ in canine SCC and TCC have not been evaluated. Possible hypotheses for these discrepant results include changes in the expression of EP4 receptors due to certain conditions in the tumor microenvironment, alterations in cell density, or differences in tissue specific functional activities known to exist for EP receptors [15, 40]. Further evaluation of additional tumor samples from throughout the urothelial tract, HIF-1⍺ levels, EP3R expression, and the tumor microenvironment will be needed to help clarify the role of EP4R in this tumor type.

Multiple canine malignancies, including cutaneous SCC, AGASACA, and TCC, have been shown to have increased COX-2 expression [24, 31, 43]. Blockade of COX-2 with NSAIDs, alone or in conjunction with chemotherapy, leads to tumor control and prolonged survival in many of these malignancies [26, 32]. However, NSAIDs are not specific and attenuate the production of prostanoids other than PGE2 that are important in homeostasis [33]. Thus, alternative treatment options that are more specific, such as an EP4R antagonist, may be clinically beneficial.

In human medicine, the impact of EP4R antagonists on cancer have been evaluated in murine models and naturally occurring malignancies. Positive results have been noted in solid tumors including prostate, breast, and lung carcinoma, colorectal carcinoma, and melanoma [9, 12]. In a cell line model of human urothelial carcinoma, EP4R antagonists decreased cancer cell migration and viability, and enhanced the effects of a commonly used chemotherapeutic, cisplatin, implying the potential role of EP4R antagonists in the treatment of several human malignancies [44]. The in vitro and in vivo effects of an EP4R antagonist on canine cancer has not been evaluated. However, the successful anti-inflammatory effects of the specific EP4R antagonist grapiprant have been proven in both murine models [33] and dogs with naturally occurring osteoarthritis [45]. In dogs, grapiprant was proven to be safe and effective in limiting inflammation and controlling pain. The information gleaned from this study suggests that if the gene expression of EP4R correlates to protein expression, contributing to the development of malignancy, blockade of EP4R with a piprant drug, such as grapiprant, may be a therapeutic approach for multiple canine tumors [33].

Statistically significant differences in the expression of EP4R were found for canine cutaneous SCC, AGASACA, and TCC. While this is exciting preliminary data, it must be interpreted in the light of a small sample size and recognition that gene expression does not necessarily correlate to significant protein expression. Similarly, confirmation that EP4R is the major isoform in canine malignancy will need to be proven with evaluation of the expression of EP1R, EP2R, and EP3R. As this represents a pilot and proof of concept study, additional tumor specimens, histologic analysis of EP4R, evaluation of EP4R protein expression, and correlation with case outcome and treatment, will be necessary to confirm these results and to make significant inferences regarding blockade of EP4R as a therapeutic strategy.

## Conclusions

The novel mRNA in situ hybridization platform, RNAscope®, was successfully used to characterize EP4R expression in three canine malignancies. This is the first reported evaluation of EP4R in veterinary medicine based on RNAscope®, and serves as a scientific basis for future evaluations of all EP receptors in both research and clinical settings.

The current study revealed positive gene expression of EP4R in three common, aggressive canine malignancies. In canine cutaneous SCC and AGASACA, higher expression of EP4R mRNA was identified when compared to normal tissues. Canine TCC was found to have less EP4R mRNA expression when compared to the normal bladder. These results suggest that EP4R may be involved in the development of certain canine cancers. If EP4R proves to be the major isoform in these tumor types, therapeutic blockade with an EP4R antagonist, such as the commercially available grapiprant, may be advantageous, changing the treatment paradigm in veterinary medicine.

## Methods

### Tumor and normal tissue samples

Archived biopsy and necropsy tissue specimens maintained by the Department of Veterinary Pathology at Iowa State University were searched for formalin-fixed, paraffin embedded tumor and normal tissue samples. Based upon preliminary data, a power calculation indicated that 9 specimens in each group would be statistically adequate to identify a difference of 2 transcript copy number/cell between tumors (SCC, AGASACA, and TCC) and respective normal tissue (skin, anal sac, and bladder mucosa) (alpha = 0.05, beta = 0.2). The institutional animal care and use committee of Iowa State University did not require prior approval for retrospective studies utilizing data and tissue specimens generated through routine clinical assessment and care of patient animals. The authors had permission from the Veterinary Teaching Hospital and Department of Veterinary Pathology to use the clinical data and samples, such that owner and patient information remains anonymous.

### EP4R mRNA expression in tumor and normal tissue samples

The RNAscope® mRNA in situ hybridization platform (Advanced Cell Diagnostics, Hayward California USA) was used to evaluate mRNA expression of EP4R gene *ptger4* in the tumor and respective normal tissue samples. A single-plex, manual chromogenic RNAscope® analysis was performed per the manufacturer’s instructions and as previously reported with an RNAscope® 2.5 High Definition (HD) – RED Assay (Catalog #322350, Advance Cell Diagnostics) [46–49]. Briefly, three sections of each paraffin embedded tissue specimen were cut to a 5 μm depth. Preparations were baked for 1 h at 60 °C, deparaffinized, and subsequently protease treated to expose RNA. The three sections were then hybridized separately with a test probe targeting canine *ptger4* (Probe CI-PTGER4, Catalog # 499011, Advanced Cell Diagnostics), a positive control probe targeting canine house-keeping gene *ubc* (CI-UBC Positive Control, Catalog # 409851, Advanced Cell Diagnostics), and a negative control probe targeting *Bacillus subtilis dapB* (DapB Negative Control, Catalog # 310043, Advance Cell Diagnostics). Unlike traditional quantitative real time polymerase chain reaction, normalization of EP4R expression to the housekeeping gene is not required [46, 47]. Hybridization to target mRNA was performed by incubating the preparation with the respective probe at 40 °C for 2 h in a HybEZ hybridization oven (Advanced Cell Diagnostics). Subsequent wash and signal amplification steps were performed according to the manufacturer’s protocol. Target mRNA was detected using alkaline phosphatase Fast Red chromogenic stain (Catalog # 320701, Advanced Cell Diagnostics). Samples were also stained with hematoxylin (American Master Technology, California USA) to permit visualization of nuclei. To initially assess the performance of the *ptger4* RNAscope® experimental probe, *ptger4* expression was assessed in two sections each of normal canine heart, lung, and kidney tissue. Expression of p*tger4* expression has been previously reported in these canine tissues [28] and was identified in all three tissues types with the RNAscope® analysis with this *ptger4* probe.

### Quantification of mRNA expression

For each hybridized slide, ten 400x magnified non-overlapping microscopic field views were digitally photographed. Digitized photomicrographs were then evaluated with the RNAscope® image analysis software HALO with the RNAscope® Modules (Indica Labs, Albuquerque New Mexico USA) [50]. For tumor samples, the neoplastic cells were manually identified and gated for analysis. For normal tissue samples, the appropriate normal cell population was manually identified and gated for analysis. Samples were considered to have adequate residual RNA for *ptger4* expression analysis if the corresponding positive control *ubc* hybridization yielded > 15 transcript dots/cell in > 90% of the target cells. The following metrics were then calculated for *ptger4* expression based upon the cumulative analysis results of the ten digitized photomicrographs for the slide: average transcript copy number/cell, percent probe positive expression (percent of cells positive for EP4R mRNA), and H-score (a weighted expression scale used to evaluate heterogeneity in marker expression; A Guide for RNAscope® Data Analysis, Advanced Cell Diagnostics). These combined techniques allow for quantification of the gene expression within a specific cell type and tissue context, and comparison of expression across tumor types.

### Statistical analysis

A D’Agostino Pearson test was used to evaluate normality of copy number per cell, H-score, and percent probe positive expression for each tumor type and normal tissues; all data was non-normally distributed. A Wilcoxon rank-sum test was used to assess differences in the aforementioned parameters between tumors and respective normal tissue types. Statistical significance was defined as *p* < 0.05. Where applicable, nonparametric data is presented as median ± range. Statistical comparisons were performed using a commercially available software package (Prism 6. GraphPad Software, Inc. San Diego CA USA). Power calculation was performed with a separate software package (MedCalc. MedCalc Software BV. Ostend, Belgium).

## Data Availability

The data that support the findings of this study are available from the corresponding author upon reasonable request.

## Abbreviations

AGASACA: Apocrine Gland Anal Sac Adenocarcinoma
COX2: Cyclooxygenase enzyme 2
EP4R: EP4 Receptor
FI: Female Intact
FS: Female Spayed
HIF-1⍺: Hypoxia inducible factor 1-alpha
MI: Male Intact
MN: Male Neutered
NR: Not Reported
PG: Prostaglandins
PGE2: Prostaglandin E2
SCC: Squamous Cell Carcinoma
TCC: Transitional Cell Carcinoma
UVB: Ultraviolet B
